# Descriptions of Phenomenal Versus Intentional Mental States Are Differentially Associated With Gaze Direction

**DOI:** 10.1162/OPMI.a.386

**Published:** 2026-09-20

**Authors:** Mohit Mukherji, Moira R. Dillon

**Affiliations:** Department of Psychology, New York University, New York, NY, USA

**Keywords:** gaze direction, mind perception, mental states, phenomenality, intentionality, social cognitive development

## Abstract

Across cultures, humans reason about others’ mental states as being either *phenomenal*, reflecting shareable experiences, or *intentional*, reflecting agentive goals. Might our dissociated judgments of others’ mental states be linked to dissociated judgments of others’ gaze direction, one of our earliest social cues from infancy? We presented adults (N = 600) and 6- to 8-year-old children (N = 200) with direct, definitional descriptions as well as descriptions of example phenomenal or intentional mental states and asked them to choose whether a face with averted or straight-ahead gaze better matched the character in each description. Adults and older children chose the averted-gaze face for the direct phenomenality description but the straight-ahead-gaze face for the direct intentionality description. Adults showed the same pattern of responses for the indirect, example descriptions, but children chose the straight-ahead-gaze face for both types of indirect descriptions. Descriptions of either phenomenal or intentional mental states might be intuitively associated with straight-ahead gaze, perhaps originating in infants’ understanding of straight-ahead gaze as communicating either mutual social engagement or general visible accessibility for goal-directed action. The association between phenomenality descriptions and averted gaze may, by contrast, arise only after learning that certain phenomenal contexts involve inner experiences with averted gaze (e.g., someone is looking at a particular emotion-evoking stimulus or looking “into their own mind”). If, in adulthood, our social concepts have indeed been built on prelinguistic intuitions that can be evoked by language, then these intuitions may continue to affect our mature, linguistically driven social reasoning.

## INTRODUCTION

The last two decades or so of research in the psychological sciences have suggested that, across cultures, we reason about descriptions of other people’s mental states as either *social*, reflecting shareable *phenomenal* experiences, or *agentive*, capturing the *intentional* pursuit of goals (e.g., Gray et al., 2007; Weisman et al., 2021). As adults, we make inferences about the content of others’ minds from such mental-state descriptions as well as from a variety of nonlinguistic cues, like others’ emotional expressions (Barrett, 2011; Ekman, 1992; Wu et al., 2018), actions (Anzellotti & Young, 2020; Wang et al., 2025), and gaze directions (Colombatto et al., 2020; Khalid et al., 2016).

Gaze direction may support flexible inferences about others’ mental states by conveying either phenomenal experiences or intentional goals. For example, while adults rate pictures of real faces with neutral expressions as more likely to reflect phenomenal mental states than intentional ones regardless of gaze direction and straight-ahead-gaze faces as reflecting more mental content overall than averted-gaze faces, mental-state attributions and gaze direction also interact: Adults rate straight-ahead versus averted-gaze faces as more likely to reflect intentional mental states, but they do not rate straight-ahead versus averted-gaze faces (or vice versa) as more likely to reflect phenomenal mental states (Khalid et al., 2016). How might such complex mental-state attributions from gaze direction emerge?

Young infants look longer at others who engage them with straight-ahead “at-me” gaze versus averted gaze (Farroni et al., 2002), and they use such mutual gaze as a social cue to share states of attention and phenomenal experiences with others (Jones, 2008; Jones et al., 1991). Infants fail, however, to recognize others’ averted gaze to an object as intentional until 12 months (Phillips et al., 2002; Woodward, 2003), and their understanding of averted, object-directed gaze undergoes protracted development. For example, not until around age 10 months do infants turn to look at an object another person turned their head towards, and this is regardless of whether that person’s eyes are open or closed (Brooks & Meltzoff, 2005). In the same situation, 12-month-olds only look when the person’s eyes are open, but they still do not take into account whether the person is wearing a blindfold or not (Brooks & Meltzoff, 2002).

Infants’ failure to use averted gaze to infer others’ object goals, moreover, is not because infants generally fail to understand others’ object goals entirely. Indeed, even young infants understand others’ object-directed actions (e.g., reaching) as both intentional and rational from early infancy (e.g., Liu et al., 2019; Stojnić et al., 2023; Woodward, 1998). And, infants recognize and rely on what is generally visibly accessible to someone when determining what their object goals could be (Luo & Johnson, 2009). Thus, in infancy, straight-ahead gaze can serve as both a social cue for mutual engagement as well as an indicator of general visual accessibility for goal-directed action. Averted gaze, by contrast, may provide infants with little to no insight into others’ phenomenal experiences or their intentional goals.

The present experiments aim to explore whether and how such early limitations to the use of gaze direction for mental-state reasoning might be related to later judgments about mental-state descriptions. If there is such a relation, then that might provide insight into the intuitive content on which our mature social concepts are built and thereby inform how we engage with the linguistic and formal systems of ethics and morality that rely on such concepts (Knobe & Prinz, 2008; Reilly, 2019).

To explore these possibilities, we first examine whether adults associate descriptions of others’ phenomenal versus intentional mental states with different judgments of the gaze direction in schematic and real faces across three experiments. Experiment 1 presents adult participants with a succession of descriptions of either phenomenal or intentional mental states, each paired with two schematic pictures of faces, one with straight-ahead gaze and one with averted gaze, faces like those used to test newborn infants’ face recognition. Adults are then asked to pick the face that best matches the character in the description. Experiment 2 provides a preregistered replication of Experiment 1, and Experiment 3 evaluates the findings’ generalizability by presenting pictures of real faces, like those used to test infants’ preferences for straight-ahead versus averted gaze.

Our first set of 18 mental-state descriptions indirectly capture phenomenal versus intentional mental content by adapting descriptions from Gray et al. (2007), who presented a large, international sample of adults with different human and nonhuman characters (e.g., a baby, a woman, a robot, a dog, etc.) and asked adults to compare those characters based on their ability to have certain kinds of mental states. Gray et al. (2007) found that adults’ judgments were best characterized by two principal components, which they named “experience” and “agency.” “Experience” descriptions reflect phenomenal mental states, like hunger, whereas “agency” descriptions reflect intentional mental states, like planning. This distinction mirrors the early dissociation infants show in their sensitivity to social beings with shareable phenomenal experiences versus agents with nonsocial goals (Spelke, 2022). We label Gray et al.’s (2007) descriptions as “indirectly” capturing phenomenal or intentional mental states because they provide specific examples of such mental states. Our second set of two mental-state descriptions “directly” capture phenomenal versus intentional mental content by providing definition-like descriptions informed by Spelke (2022), a seminal review of infants’ social cognitive abilities. These direct descriptions explicitly describe social beings as having shareable phenomenal experiences versus agents as having intentional goals.

Our work also builds on the prior work reviewed above by Khalid et al. (2016). In their study, adults saw pictures of real faces with neutral expressions displaying either straight-ahead or averted gaze and were asked to rate on a 7-point Likert scale the extent to which each face expressed a phenomenal or intentional mental state. They used the eight “experience” and “agency” mental-state descriptions from Gray et al. (2007) that had the highest factor loadings (the highest four descriptions from each dimension). Based on their finding that adults rate faces with straight-ahead versus averted gaze as more likely to reflect intentional mental states, Khalid et al. (2016) concluded that another person’s straight-ahead gaze communicates their likelihood to approach as well as what they might do in an engagement. Because these differential judgments about gaze direction were not present for phenomenal mental states, Khalid et al. (2016) suggested that it may be relatively less important to attend to a person’s internal experiences when making predictions about their potential approach or actions in an engagement. We believe these interpretations nevertheless raise questions about why the inner experiences described by the phenomenality mental-state descriptions should not motivate approach or why judgments of gaze direction should be limited to predictions about a person’s likelihood to approach to begin with.

In the present work, we thus build on this prior work in several ways to offer an interpretation of their prior findings as well as our present findings. To do so, we first include the full set of Gray et al. (2007) descriptions as well as the direct, definitional versions of Gray et al.’s (2007) dimensions inspired by Spelke (2022). Khalid et al. (2016) used only a subset of Gray et al.’s (2007) mental-state descriptions, in particular, ones that described drive states (e.g., hunger) or basic emotions (e.g., fear), and so our complete set of descriptions may more comprehensively engage dissociated judgments of gaze direction. Second, we use a simple two-alternative forced choice task, in which participants are asked to explicitly choose between two faces differing in gaze direction. This design aims to eliminate any ambiguity about what the task is targeting. Moreover, while we are unlikely to encounter such a task or such decontextualized faces with specific gaze directions in our everyday lives, their use in the present experiments provides insight into what social information we might encode automatically and intuitively, potentially revealing the otherwise hidden foundations of our social reasoning or what we might fall back on in our social judgments when this reasoning does happen in context. If such decontextualized stimuli can engage no such “defaults,” however, then we would expect no consistent patterns of responding. Third, we use compelling face stimuli that have even been shown to engage the social intuitions of young infants (Farroni et al., 2002, 2004). In doing so, moreover, we can begin to consider adults’ responses relative to the responses of infants in prior studies.

We also examine the development of the relation between gaze direction and mental-state descriptions in Experiment 4, which adapts Experiment 2 for 6- to 8-year-old children. Children in this age range are just beginning to show adult-like judgments about descriptions of others’ mental-state capacities (Gray et al., 2007; Weisman et al., 2018), while their understanding of mental-state language (Baron-Cohen et al., 2010) and their mental-state and theory-of-mind reasoning (Grazzani & Ornaghi, 2012; Harris et al., 2005; Pons et al., 2003) are also undergoing substantial development. Testing children at these ages will thereby allow us to assess whether associations between gaze direction and mental-state descriptions emerge over the same developmental time window as these other abilities and whether younger children associate both phenomenal and intentional mental-state descriptions with straight-ahead gaze, consistent with infants’ use of straight-ahead gaze to indicate both phenomenal and intentional mental states.

We predicted that our use of direct, definitional descriptions would yield more robust and perhaps earlier emerging dissociated judgments of gaze direction compared with the indirect, example descriptions. We also predicted that the combination of our comprehensive set of descriptions, straightforward methodology, and the use of face stimuli from the infancy literature would lead to dissociated judgments of gaze direction for both the intentionality descriptions as well as the phenomenality descriptions, although the dissociation might be stronger and/or earlier emerging for the intentionality descriptions, following prior work. Finally, we predicted that there would be developmental change in children’s dissociated judgments of gaze direction for different mental-state descriptions, perhaps due to age-related changes in judgments of gaze direction and/or understanding of mental-state language.

## EXPERIMENT 1

### Methods

#### Participants.

Assuming a medium effect size of *f* = 0.3, we conducted a power analysis with G*Power (Erdfelder et al., 1996) for a repeated-measures, within-participants ANOVA aimed at determining whether there could be a difference in participants’ choice of gaze direction for the two description types. We found that N = 200 participants would lead to .85–.90 power with this analysis.

N = 200 English-speaking adults (185 native speakers, i.e., learned before age 5 years; 15 nonnative speakers) recruited through our university’s study pool constituted our sample (124 female, 74 male, 2 nonbinary; *M*_*age*_ = 19.69 years; *range* = 18–23 years). Seventeen additional adults participated but were excluded by a masked experimenter for: not completing the experiment (6); choosing the same face more than 90% of the time (4); and taking more than 15 minutes to complete the experiment (7). The experiment was approved by our university’s institutional review board on the use of human participants.

#### Materials.

On separate trials, participants read the 18 descriptions of characters adapted from Gray et al. (2007). Eleven of the descriptions were better captured by Gray et al.’s (2007) “experience” dimension (e.g., “This character is capable of feeling hungry.”), and seven were better captured by Gray et al.’s (2007) “agency” dimension (e.g., “This character is capable of making plans and working towards goals.”) (see the Supplementary Information for all 18 descriptions). We called these descriptions the “indirect phenomenality descriptions” and the “indirect intentionality descriptions,” as described in the Introduction. Participants then read the two descriptions adapted from Spelke (2022), one which aimed to explicitly capture the idea of a phenomenal being (“I am a social being. I have emotions and feelings like you do.”) and one which aimed to explicitly capture the idea of an intentional agent (“I am an agent. I have goals and intentions that I pursue efficiently.”). We called these descriptions the “direct phenomenality description” and the “direct intentionality description,” as described in the Introduction.

Accompanying the written text description for each trial was a picture of two schematic faces (Figure 1), each presenting just a circle as a head and two eyes, each composed of two circles, inspired by the face stimuli used to test newborn infants’ face recognition (Farroni et al., 2004). The face on the left always depicted straight-ahead gaze, and the face on the right always depicted averted gaze.

**Figure 1.**
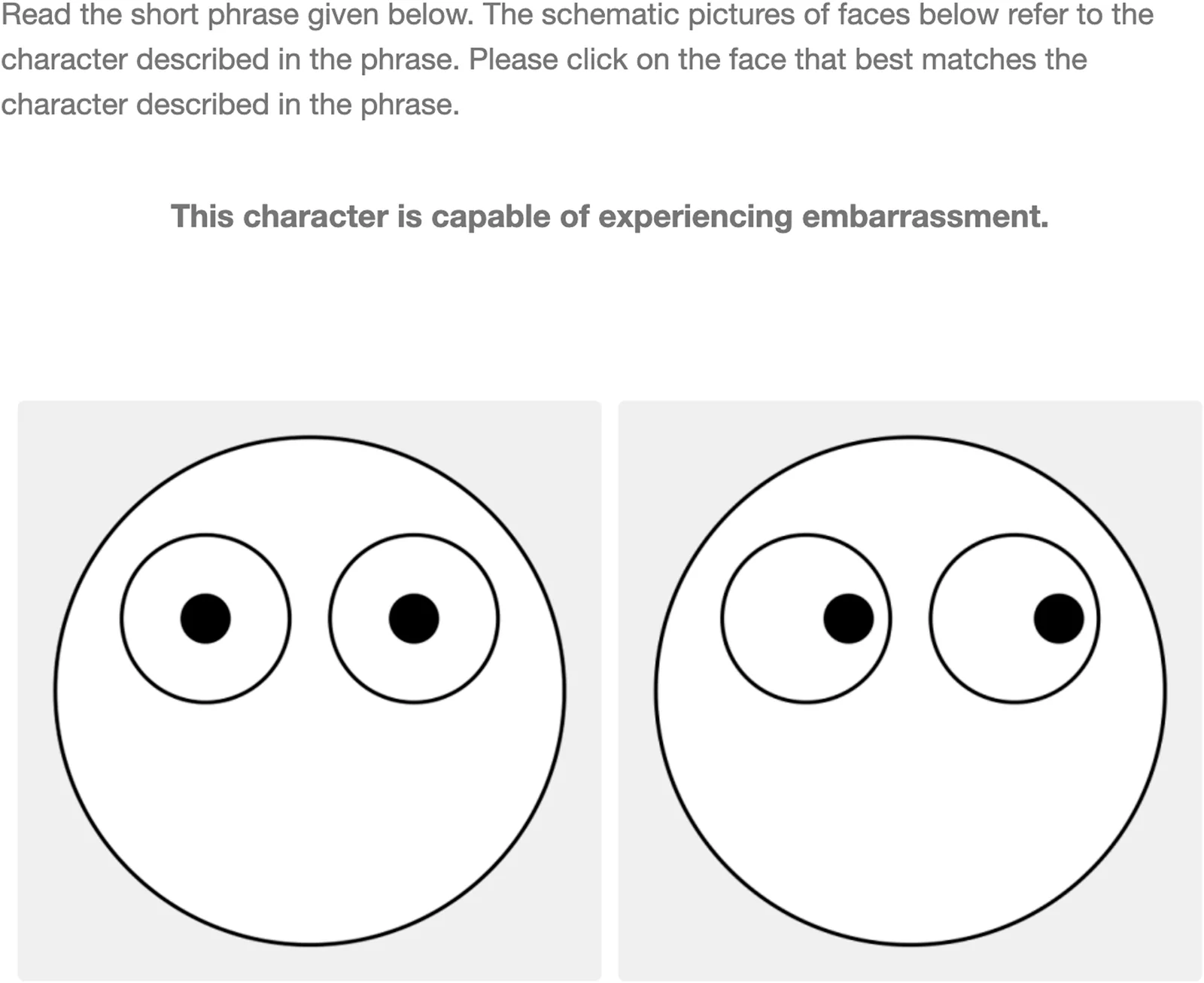
Example experimental display used in Experiments 1, 2, and 4 (with faces stimuli after Farroni et al., 2004). In Experiment 1, the face on the left always depicted straight-ahead gaze, and the face on the right always depicted averted gaze, but in the other experiments, the side of each face was randomly assigned. Children in Experiment 4 saw the same displays as adults in Experiment 2 but heard the instructions and descriptions read aloud by a live experimenter.

#### Procedure.

Participants completed the experiment online and asynchronously on Qualtrics, following the written instructions. Participants first saw a screen where they adjusted the zoom level on their browser window so that they could see a description as well as both schematic faces on their screen without scrolling. Participants were then told that they would read some descriptions of characters and that each of the faces below the description could refer to the character being described. For each description, participants were asked to pick the face that best matched the character in the description (Figure 1). In the first block of 18 trials, participants responded to the indirect descriptions in a random order, and in the second block of 2 trials, participants responded to the direct descriptions in a random order. Participants were given no feedback.

### Results

Even though our power analysis was based on a mixed-design ANOVA, we opted for mixed-effects regressions where appropriate so that we could model random effects and better account for any potential dependencies in our data. To obtain *p*-values from the regressions, we ran Type 3 Wald tests on the results. Where appropriate, we also report the models’ estimates as proportions of responses (P) with 95% confidence intervals.

The first two models evaluated adults’ choices against chance for the indirect descriptions using binomial mixed-model logistic regressions with the gaze direction participants chose (straight-ahead or averted) as the dependent variable. Adults chose more faces with averted gaze for the indirect phenomenality descriptions (P = .55, 95% CI [.53, .58]; *χ*^2^(1) = 19.73, *p* < .001) but more faces with straight-ahead gaze for the indirect intentionality descriptions (P = .57, 95% CI [.53, .60]; *χ*^2^(1) = 12.45, *p* < .001; Figure 2). A third model including description type as a fixed effect found that adults’ choices differed based on description type (*χ*^2^(1) = 44.57, *p* < .001).

**Figure 2.**
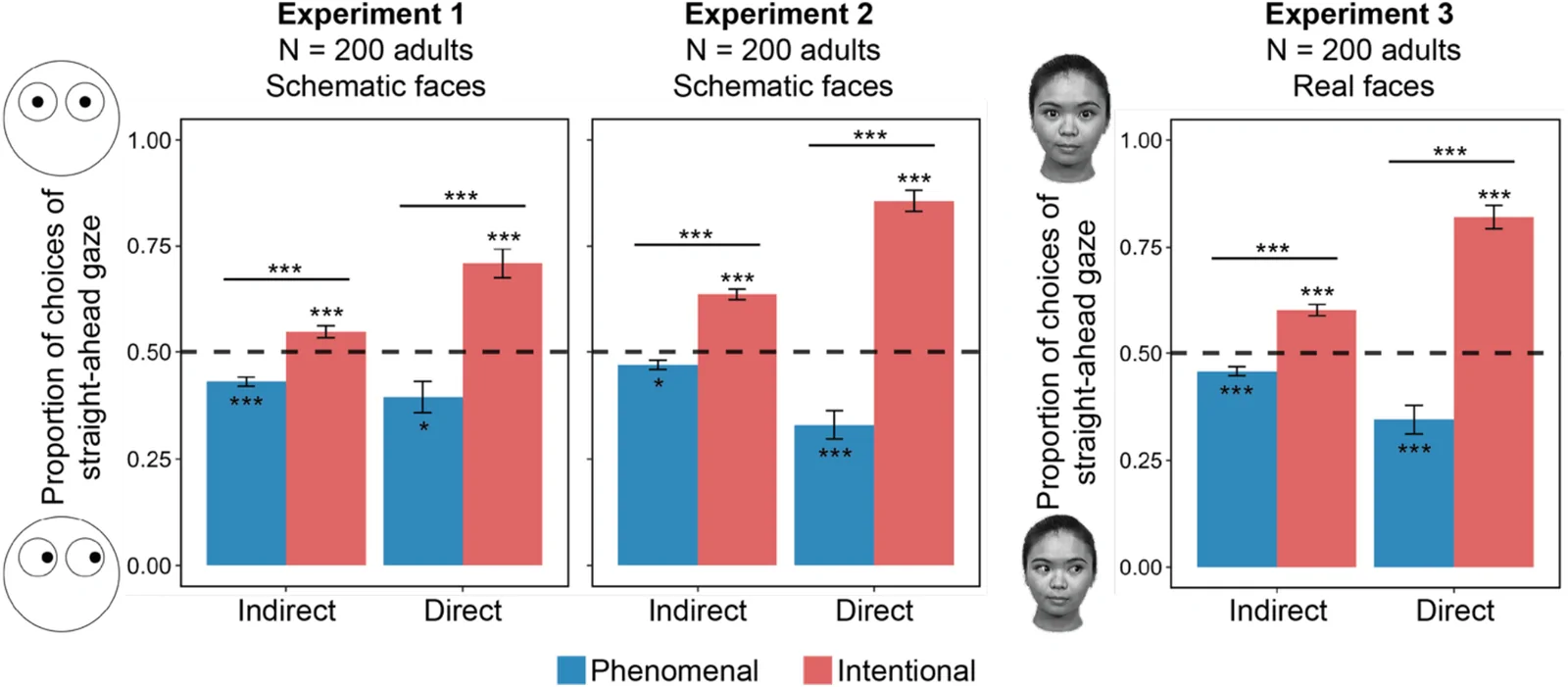
Results of Experiments 1, 2, and 3 with adults. Adults’ responses to the indirect descriptions from Gray et al. (2007) are presented on the left of each experiment’s graph, with phenomenality descriptions in blue and intentionality descriptions in red. Adults’ responses to the direct descriptions from Spelke (2022) are presented on the right side of each experiment’s graph with the same color coding. The error bars present standard errors of the mean. *p < 0.05, **p < 0.01, ***p < .001. © 2002 National Academy of Sciences. All rights reserved.

Because Gray et al. (2007) reported each description’s factor loadings on their experience and agency dimensions on a continuous scale, the next two models used linear regressions to evaluate whether the frequency of adults’ choice of gaze direction was related to the magnitude of these factor loadings. For each kind of description, we included factor loading as the dependent variable, adults’ choices as a fixed effect, and participant as a random-effects intercept. We found that the frequency of adults’ choices related to the magnitude of the Gray et al. (2007) factor loadings for both the phenomenality (*F*[1, 2198] = 8.73, *p* = .003; Figure S1A) and intentionality descriptions (*F*[1, 1398] = 24.72, *p* < .001; Figure S1B).

Next, we ran two binomial tests to evaluate adults’ choices against chance for the direct descriptions and compared their choices across the two kinds of descriptions using a binomial mixed-model logistic regression. Consistent with the results from the indirect descriptions, adults chose more faces with averted gaze for the direct phenomenality description (58%, *p* = .028) but more faces with straight-ahead gaze for the direct intentionality description (68%, *p* < .001; Figure 2), and adults’ choices differed by description type (*χ*^2^(1) = 26.64, *p* < .001).

Finally, we compared across the indirect and direct descriptions using a binomial mixed-model logistic regression that included description type, the directness of the description, and their interaction as fixed effects. We found main effects of description type (*χ*^2^(1) = 28.08, *p* < .001) and the directness of the description (*χ*^2^(1) = 10.83, *p* = .001). Moreover, we found a description type by directness interaction (*χ*^2^(1) = 8.57, *p* = .003), with adults showing a stronger dissociation in their judgments of gaze direction based on description type.

## EXPERIMENT 2

Experiment 2 was a preregistered replication and extension of Experiment 1.

### Methods

#### Participants.

We conducted a power analysis by bootstrapping our data from Experiment 1 along with an additional set of participants we had collected earlier (N = 37; total N = 237), extracting 1,000 samples with replacement and testing whether the analytic models we ran in Experiment 1 were statistically significant. We found that a sample size of N = 200 led to significant outcomes > .95 of the time and preregistered that sample size prior to data collection.

N = 200 native English-speaking adults recruited through our university’s study pool constituted our sample (147 female, 50 male, 3 nonbinary; *M*_*age*_ = 19.40 years, *range* = 18–24 years). An additional 58 adults participated but were excluded by a masked experimenter based on our preregistered criteria for: not completing the experiment (7); choosing the same face more than 90% of the time (4); taking more than 15 minutes to complete the experiment (23); or failing an attention-check question (24; see below). While this exclusion rate was higher than that of Experiment 1, it is consistent with those of other unmonitored online experiments for adults using similar attention-check questions (which was new to Experiment 2) to ensure data quality (e.g., Lin & Dillon, 2024; Ludwin-Peery et al., 2020). The experiment was approved by our university’s institutional review board on the use of human participants.

#### Materials.

The materials were identical to those of Experiment 1 except that we changed the direct descriptions from first person (“I am a…”) to third person (“This character is a…”) to match the format of the indirect descriptions. In addition, unlike Experiment 1, the straight-ahead- and averted-gaze faces in Experiment 2 could appear on either the left or right side of the screen. The averted-gaze face looked to the left when it was presented on the left and to the right when it was presented on the right (so that the averted-gaze face always looked away from the straight-ahead-gaze face). The side on which each face was presented was randomized across trials and across participants.

#### Procedure.

The procedure was identical to that of Experiment 1 except that we added an attention-check question after the experience description describing hunger. Participants were asked, “What was the character described on the previous page capable of?” and they were given the choice of “feeling tired,” “feeling thirsty,” “feeling hungry,” or “feeling shameful” in this fixed order. They received no feedback on their response.

### Results

Our analyses were identical to those of Experiment 1 and were preregistered prior to data collection unless otherwise specified.

As in Experiment 1, adults chose more faces with averted gaze for the indirect phenomenality descriptions (P = .53, 95% CI [.51, .56]; *χ*^2^(1) = 5.94, *p* = .015) but more faces with straight-ahead gaze for the indirect intentionality descriptions (P = .66, 95% CI [.62, .70]; *χ*^2^(1) = 50.84, *p* < .001; Figure 2), and participants’ responses differed by description type (*χ*^2^(1) = 96.73, *p* < .001). The frequency of adults’ choices of straight-ahead or averted gaze related to the magnitude of the Gray et al. (2007) factor loadings for the intentionality descriptions (*F*[1, 1398] = 41.07, *p* < .001; Figure S2A), but not for the phenomenality descriptions (*F*[1, 2198] = 0.57, *p* = .451; Figure S2B).

Consistent with the results from the indirect descriptions and with the results from Experiment 1, adults chose more faces with averted gaze for the direct phenomenality description (67%, *p* < .001) but more faces with straight-ahead gaze for the direct intentionality description (86%, *p* < .001; Figure 2), and adults’ choices differed by description type (*χ*^2^(1) = 54.76, *p* < .001). Finally, and again consistent with the results from Experiment 1, a post-hoc analysis revealed that adults’ dissociated use of gaze was stronger for the direct versus indirect descriptions (*χ*^2^(1) = 50.51, *p* < .001).

## EXPERIMENT 3

Experiment 3 was a preregistered extension of Experiments 1 and 2. Instead of using schematic faces, like the ones used to test infants’ face recognition (Farroni et al., 2004), Experiment 3 used real pictures of faces, like the ones used to test infants’ preferences for direct versus averted gaze (Farroni et al., 2002). The use of real faces tests the generalizability of adults’ judgments about the relation between others’ mental states and their gaze direction across schematic and real faces.

### Methods

#### Participants.

The sample size was chosen to match the prior experiments and was preregistered prior to data collection. N = 200 native English-speaking adults recruited through our university’s study pool constituted our sample (127 female, 69 male, 4 nonbinary; *M*_*age*_ = 19.51 years, *range* = 18–24 years). An additional 82 adults participated but were excluded by a masked experimenter for: not completing the experiment (6); choosing the same face more than 90% of the time (12); taking more than 15 minutes to complete the experiment (29) or for failing the catch question (35). The experiment was approved by our university’s institutional review board on the use of human participants.

#### Materials.

The materials were identical to those of Experiment 2 except that we replaced the schematic faces with pictures of real faces with neutral expressions (Figure 3). Farroni et al. (2002) had used versions of these faces to test newborn infants’ looking-time preferences to faces with straight-ahead versus averted gaze.

**Figure 3.**
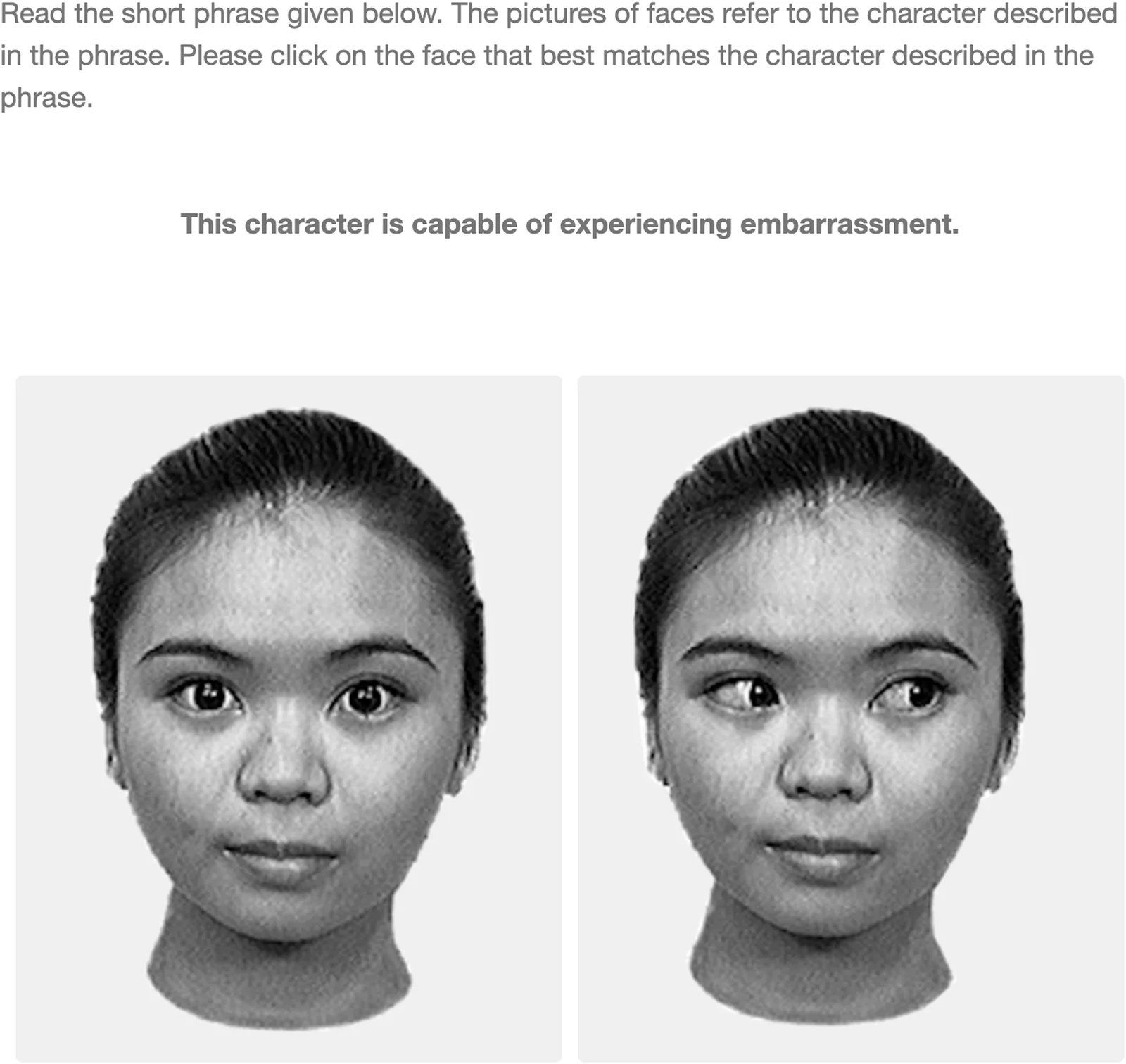
The face stimuli and a sample question from Experiment 3, which presented adult participants with pictures of real faces from Farroni et al. (2002). © 2002 National Academy of Sciences. All rights reserved.

#### Procedure.

The procedure was identical to that of Experiment 2 except that we added two “debriefing” questions at the end that explored participants’ interpretation of the two faces’ gaze directions. For each question, participants were presented with one of the faces in a random order and asked, “Where does it seem like this character is looking?” Participants were given the choice of “at a particular object, person, or place,” “at anything in front of them,” “at me,” or “other.”

### Results

All analyses were preregistered prior to data collection unless otherwise specified. The initial models were identical to those of Experiments 1 and 2. In addition, we specified two binomial mixed-model logistic regressions comparing the results across experiments to evaluate whether adults’ responses to the real faces in Experiment 3 differed from adults’ responses to the schematic faces in Experiment 2.

As in Experiments 1 and 2, adults chose more faces with averted gaze for the indirect phenomenality descriptions (P = .54, 95% CI [.52, .57]; *χ*^2^(1) = 12.51, *p* < .001) but more faces with straight-ahead gaze for the indirect intentionality descriptions (P = .61, 95% CI [.58, .65]; *χ*^2^(1) = 37.81, *p* < .001; Figure 2), and adults’ responses differed by description type (*χ*^2^(1) = 73.21, *p* < .001). As in prior experiments, the frequency of adults’ choices of straight-ahead or averted gaze related to the magnitude of the Gray et al. (2007) factor loadings for both the phenomenality descriptions (*F*[1, 2198] = 12.31, *p* < .001; Figure S3A) and the intentionality descriptions (*F*[1, 1398] = 21.92, *p* < .001; Figure S3B).

Consistent with the results from the indirect descriptions and with the results from Experiments 1 and 2, adults chose more faces with averted gaze for the direct phenomenality description (66%, *p* < .001) but more faces with straight-ahead gaze for the direct intentionality description (82%, *p* < .001; Figure 2), and adults’ choices differed by description type (*χ*^2^(1) = 83.12, *p* < .001). As in prior experiments, a post-hoc analysis also found that adults’ dissociated use of gaze was stronger for the direct versus indirect descriptions (*χ*^2^(1) = 42.80, *p* < .001).

Next, we compared participants’ responses to the real faces in Experiment 3 with their responses to the schematic faces in Experiment 2 for both the indirect and direct descriptions using binomial mixed-model logistic regressions including description type, experiment, and their interaction as fixed effects. For the indirect descriptions, we found a main effect of description type (*χ*^2^(1) = 73.52, *p* < .001), but no main effect of experiment (*χ*^2^(1) = 2.76, *p* = .096). We also found no interaction between description type and experiment (*χ*^2^(1) = 0.93, *p* = .335), providing no evidence that adults’ responses to the real faces in Experiment 3 were different from adults’ responses to the schematic faces in Experiment 2. For the direct descriptions, we found similar results: a main effect of description type (*χ*^2^(1) = 66.73, *p* < .001) but no main effect of experiment (*χ*^2^(1) = .90, *p* = .344) and no interaction between description type and experiment (*χ*^2^(1) = .90, *p* = .344).

Finally, we conducted a post-hoc analysis of adults’ judgments of the direction of the face stimuli’s gaze (Figure S4). Notably, a greater proportion of adults judged the straight-ahead-gaze face as looking “at me” (*χ*^2^(1) = 132, *p* < .001) or “at anything in front of them” (*χ*^2^(1) = 39.77, *p* < .001) compared with the averted-gaze face, but a greater proportion of adults judged the averted-gaze face as looking “at a particular object, person, or place” (*χ*^2^(1) = 147.44, *p* < .001) compared with the straight-ahead-gaze face. In addition, adults who judged the straight-ahead-gaze face as looking at “at anything in front of them” or “at me” showed similar patterns of responding; although, those who judged the straight-ahead-gaze face as looking “at me” also showed a greater tendency to choose the straight-ahead gaze face for the phenomenality descriptions as well (*t*(1262.2) = −2.32, *p* = .021). Finally, adults who chose “other” for the averted-gaze face converged on an interpretation that was not provided as a response option but was perhaps indicative of having some kind of inner experience. They wrote in responses like, “at nothing in particular, but just looking away,” and “It doesn’t look like they are looking at something particular but more so they are having a thought.” While suggestive about participants’ interpretations of the task’s stimuli, these results are nevertheless tentative since any judgment of what a face is looking at, even when it is looking in one direction, might differ when the task is to apply a description to a face (as in our main task) versus a face to a description (as in these debriefing questions).

### Discussion

Across three experiments, adults consistently associated descriptions of phenomenal mental states with averted gaze but descriptions of intentional mental states with straight-ahead gaze. The link between intentionality descriptions and straight-ahead gaze was nevertheless stronger than the link between phenomenality descriptions and averted gaze (especially for the indirect descriptions). This finding runs in partial contrast to prior findings (Khalid et al., 2016), which found a link between judgments of gaze direction and intentionality descriptions only. Prior conclusions that gaze direction is used primarily as a cue to infer agentive mental states to predict others’ approach behavior may thus be too limited.

If adults’ judgments about gaze direction are used for more than predictions about others’ possible approach behavior, then what might they predict, in particular, about others’ mental lives? The literature reviewed above on infants’ intuitive use of gaze direction together with adults’ explicit interpretations of Experiment 3’s gaze stimuli may provide some insights. Recall that for infants, straight-ahead gaze may serve as either a social cue for shared engagement (Jones, 2008; Jones et al., 1991) or an intentional cue for general visual accessibility (Luo & Johnson, 2009). Consistent with this idea, adults who judged that the straight-ahead gaze face was looking at them (“at me”) were more likely than other adults to associate the straight-ahead-gaze face with the phenomenality descriptions. Like infants, moreover, general visual accessibility also supports adults’ judgements of others’ goals and intentions (Baker et al., 2017), and adults in Experiment 3 judged our straight-ahead-gaze face as better conveying the idea of general visual accessibility compared with our averted-gaze face.

Averted gaze, by contrast, appears to provide infants with little-to-no insight into others’ phenomenal experiences or intentional goals. Why, then, might adults link descriptions of phenomenal mental states to averted gaze? One possibility is that phenomenal mental states might initially also be linked to straight-ahead “at me” gaze, but the strength or generalizability of that link might get revised through development given experience with particular phenomenal contexts. The phenomenality descriptions may have evoked such specific ideas about a character’s inner experience that our adult participants have come to understand as better linked to averted gaze—e.g., the character might have been looking at a “particular object, person, or place” associated with the described inner experience, or they might have been “looking elsewhere” or “looking into their own mind” to feel that experience. Such interpretations may have been especially likely with our stimuli given that there was nothing else present in the pictures but the faces themselves. Future experiments might thus explore whether presenting phenomenality descriptions with averted-gaze faces directed at a specific object in the picture might affect participants’ choices of averted-gaze faces and/or increase choices of averted-gaze faces for intentionality descriptions (e.g., if these objects indicated a particular object goal or elicited a particular emotion). This interpretation might also explain why the link between the descriptions of phenomenal mental states and averted gaze was relatively weaker than the link between descriptions of intentional mental states and straight-ahead gaze.

One prediction that this interpretation makes is that children might still be associating both phenomenal and intentional mental-state descriptions with straight-ahead gaze, consistent with infants’ use of straight-ahead gaze as indicating both phenomenal and intentional mental states. As children come to learn particular examples of others’ inner experiences that are better associated with averted gaze, then they might then start shifting towards the adult-like pattern. This link between phenomenal descriptions and averted gaze might also rely at least somewhat on children’s developing understanding of the mental-state language that can capture and describe such phenomenal contexts explicitly. We test these possibilities in Experiment 4 by examining whether 6- to 8-year-old children have similar associations between gaze direction and descriptions of mental states as adults.

## EXPERIMENT 4

### Methods

#### Participants.

The sample size was chosen in advance to match the prior experiments and was preregistered prior to data collection. N = 200 native English-speaking 6- to 8-year-old children (99 girls, 101 boys; *M*_*age*_ = 7.50; *range* = 6.01–8.99) were recruited at a local museum or from our lab’s database of families interested in participating in research. An additional 12 children completed the experiment but were excluded by a masked experimenter based on preregistered criteria, including: equipment malfunction (1); experimenter error (1); parental interference (1); making a response before the experimenter delivered the prompt (4); choosing more than one face for the same trial (1); choosing the same face more than 90% of the time (2); or stopping the study early (2). The experiment was approved by our university’s institutional review board on the use of human participants.

#### Materials.

To maintain consistency with Experiments 1–3 and allow for direct comparisons between adults’ and children’s responses, the materials were identical to those of Experiment 2 except that they were presented on an iPad with a live experimenter, who read the instructions and descriptions out loud. Moreover, to confirm whether children could engage with the task’s language, we collected pilot data with a separate group of children, presenting them with each description and asking them to pick among multiple possible meanings or “glosses” for that description. Overall, children mostly chose the correct glosses. Nevertheless, we also used these pilot data to prepare a full set of 20 glosses with simplified language (the full list of glosses is provided in the Supplementary Information as is the tally of children’s gloss use). Children were encouraged to request glosses for any description during the experiment (see *Procedure* below).

#### Procedure.

Children participated in a quiet room at the museum or in the lab. They were told that they would play a quick game on an iPad, during which the experimenter would read them some descriptions of characters. They were then told that for each description, they would see two faces on the screen, and they should tap on the face that they thought best matched the character in the description. Finally, they were told that there were no right or wrong answers, and in case they were not sure what a description meant, they could ask the experimenter to say the description in a different way. If children made this request, the experimenter provided the description’s prespecified gloss, as described above.

### Results

All analyses were preregistered prior to data collection unless otherwise specified. The initial models were identical to those of prior experiments. In addition, we specified two binomial mixed-model logistic regressions comparing the results across experiments to evaluate whether children’s responses in Experiment 4 differed from adults’ responses in Experiment 2.

Unlike adults, children chose more faces with straight-ahead gaze for both the indirect phenomenality descriptions (P = .54, 95% CI [.52, .56]; *χ*^2^(1) = 12.50, *p* < .001) and the indirect intentionality descriptions (P = .53, 95% CI [.51, .56]; *χ*^2^(1) = 5.56, *p* = .018; Figure 4), and children’s responses did not differ by description type (*χ*^2^(1) = 0.06, *p* = .807). However, consistent with adults, the frequency of children’s choices of straight-ahead or averted gaze related to the magnitude of the Gray et al. (2007) factor loadings for both the phenomenality descriptions (*F*[1, 2198] = 15.42, *p* < .001; Figure S5A) and the intentionality descriptions (*F*[1, 1398] = 12.10, *p* < .001; Figure S5B).

**Figure 4.**
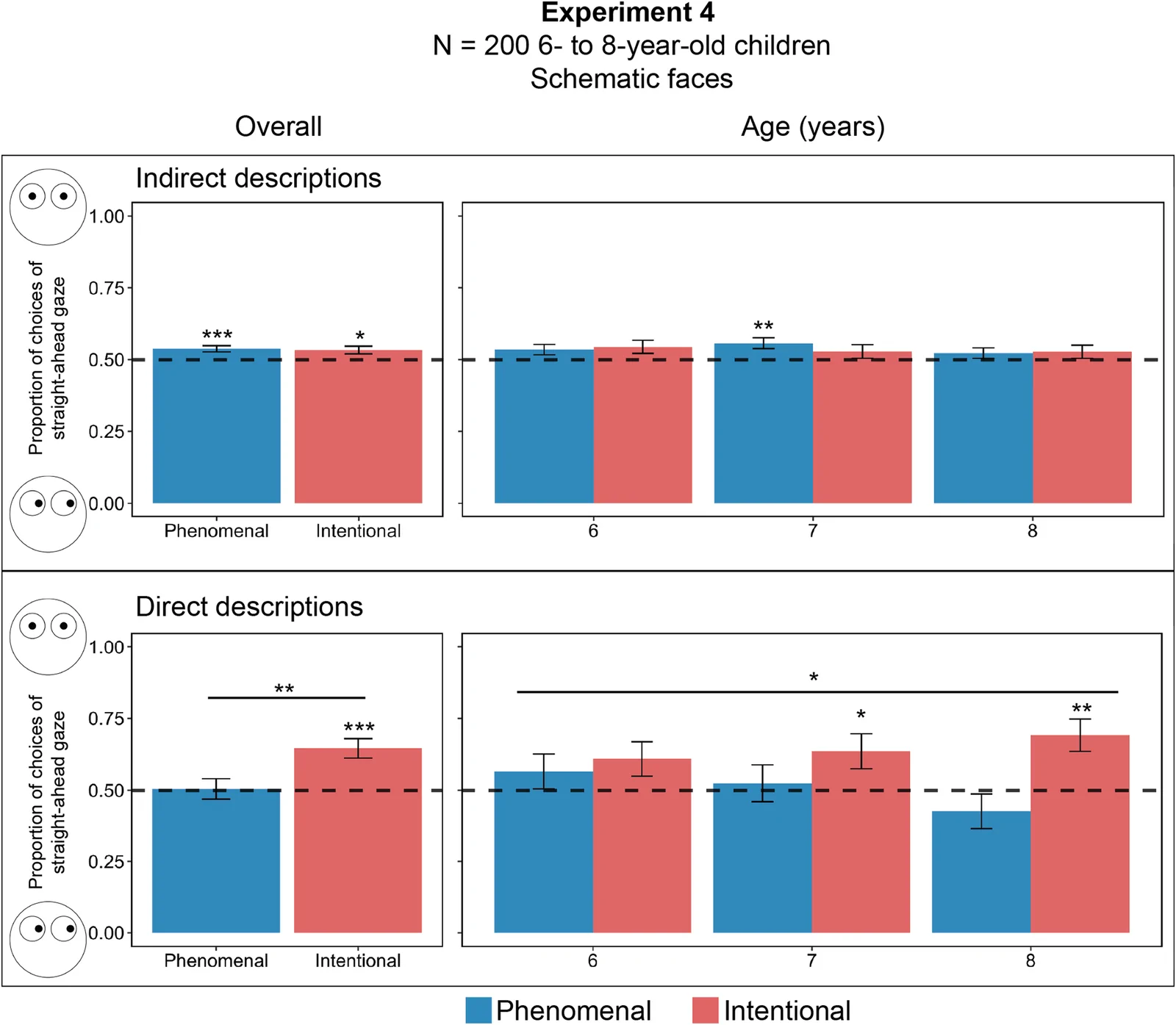
Results of Experiment 4 with 6- to 8-year-old children. Children’s responses to the indirect descriptions from Gray et al. (2007) are presented in the top panel, and children’s responses to the direct descriptions from Spelke (2022) are presented in the bottom panel. For all graphs, phenomenality descriptions are shown in blue and intentionality descriptions are shown in red. On the left of each set of graphs are children’s responses collapsed across age, and on the right of each set of graphs are children’s responses by age in years. Age was analyzed as a continuous variable in days but is shown here by year for display purposes. In addition, here we show post-hoc tests per year to indicate whether children’s responses in each age group differed from chance. Error bars represent standard errors of the mean. *p < 0.05, **p < 0.01, ***p < .001.

Unlike adults, moreover, children’s choice of faces for the direct phenomenality descriptions did not differ from chance (50%, *p* = .944), but, consistent with adults, they did choose more faces with straight-ahead gaze for the direct intentionality descriptions (65%, *p* < .001), and their choices differed by description type (*χ*^2^(1) = 7.96, *p* = .005). A post-hoc analysis revealed that children’s dissociated choice of gaze direction based on description type was stronger for the direct descriptions than for the indirect ones (*χ*^2^(1) = 7.58, *p* = .006), like it was with adults.

To better understand these results as well as determine if there was any developmental change across the child age range, we conducted a post-hoc analysis adding age (measured continuously in days) and its interaction with description type as fixed effects to our regressions. For the indirect descriptions, we found no main effect of age (*χ*^2^(1) = 1.19, *p* = .276), no main effect of description type (*χ*^2^(1) = .06, *p* = .809), and no interaction between age and description type (*χ*^2^(1) = .51, *p* = .474). For the direct descriptions, we found no main effect of age (*χ*^2^(1) = 1.69, *p* = .194), but a main effect of description type (*χ*^2^(1) = 8.00, *p* = .004). Notably, we also found an interaction between age and description type (*χ*^2^(1) = 4.47, *p* = .035), with older children choosing the averted-gaze face more for the phenomenality description but the straight-ahead-gaze face more for the intentionality description (Figure 4), consistent with adults.

To directly compare children and adults’ performance, we ran a binomial mixed-model logistic regression including description type, age group (children versus adults, treated as a categorical variable), and their interaction as fixed effects. For the indirect descriptions, we found a main effect of description type (*χ*^2^(1) = 93.77, *p* < .001) and a main effect of age group (*χ*^2^(1) = 28.91, *p* < .001). We also found an interaction between description type and age group (*χ*^2^(1) = 50.37, *p* < .001), with adults choosing the averted-gaze face more for the phenomenality descriptions but the straight-ahead-gaze face more for the intentionality descriptions. The results for the direct descriptions showed the same patterns, including main effects of description type (*χ*^2^(1) = 97.91, *p* < .001) and age group (*χ*^2^(1) = 22.29, *p* < .001) as well as an interaction between description type and age group (*χ*^2^(1) = 34.65, *p* < .001).

Finally, to better understand why children performed differently from adults, especially with the phenomenality descriptions, we performed a post-hoc analysis of children’s request for glosses as well as the difficulty of the descriptions’ language. While there was little evidence of any difference in children’s request for glosses across the two description types (see the Supplementary Information), children may have hesitated to make such requests, and so request frequency may not be the best indicator of children’s understanding of the task’s language. For a more objective measure, we analyzed each description using a standard measure of readability, the Flesch score (Flesch, 1948), using the R package quanteda (Benoit et al., 2018), with lower scores indicating more difficult language. The indirect phenomenality descriptions received a mean score of 22 (*SD* = 25.1), which is in the range of college-graduate-level readability, while the indirect intentionality descriptions received a mean score of 43.5 (*SD* = 20.1), which is in the range of college-level readability. An independent samples *t*-test suggested that the two sets of descriptions might differ in their readability (*t*(15) = 2.00, *p* = .064). The direct descriptions both received higher scores, indicating easier readability: 65.7 for the direct phenomenality description and 46.6 for the direct intentionality description.

### Discussion

Unlike adults, children chose the straight-ahead-gaze face more for both the indirect phenomenality and indirect intentionality descriptions, and this effect did not change with age. Nevertheless, like adults, their choices related to the degree to which each description captured each kind of mental state, suggesting that there was at least some differentiation in children’s responses to the two description types.

For the direct descriptions, children chose the straight-ahead-gaze face more for the intentionality description, but they chose at chance for the direct phenomenality description. This latter effect did change with age: The oldest children showed adult-like choices of the averted-gaze face for the direct phenomenality description.

These results are consistent with the interpretation we raised above that children might indeed be linking both phenomenal and intentional mental state descriptions to straight-ahead gaze, like infants. Further, children might not yet have learned that certain phenomenal contexts are better linked to averted gaze and/or their demonstration of that learning might also rely on other development, like their developing understanding of mental-state language, which continues into late childhood and early adolescence (Baron-Cohen et al., 2010; Grazzani & Ornaghi, 2012; Harris et al., 2005; Pons et al., 2003). Although we do not have the measures to evaluate whether individual children’s understanding of the tasks’ vocabulary was related to their individual patterns of responding, the language of the direct phenomenality description may have been the easiest to understand while the language of the indirect phenomenality descriptions was the hardest, and the former is where children began to show adult-like patterns. The language of the indirect phenomenality descriptions, which communicated specific inner experiences, contexts, or causes that link to averted gaze for adults, may have been out of reach for the children at the ages we tested them. Finally, as described in the Introduction, we are unlikely to encounter such a task or such decontextualized faces in our everyday lives. While adults may still be able to engage their intuitions about gaze direction and mental states in such a task/context, children’s intuitions or access to those intuitions may not be robust enough to produce significant group-wise trends without more context or other converging cues.

## GENERAL DISCUSSION

Across four experiments, we presented adult and child participants with brief descriptions of either phenomenal or intentional mental states, adapted from prior work on adult mind perception (Gray et al., 2007) and infant social cognitive development (Spelke, 2022). For each description, we asked participants to choose which of two faces, either one with averted gaze or one with straight-ahead gaze, better matched the character in each description. For both schematic and real faces as well as for both indirect, example descriptions and direct, definitional descriptions, adults chose the averted-gaze face for the phenomenality descriptions but the straight-ahead-gaze face for the intentionality descriptions. The links were nevertheless stronger for the direct versus indirect descriptions and for the intentionality descriptions and straight-ahead gaze versus the phenomenality descriptions and averted gaze.

Since we found reliable gaze-direction judgments in adults for both the phenomenality and intentionality descriptions, our findings partially contrast with those of Khalid et al. (2016), who reported links between gaze direction and intentionality descriptions only. This difference may reflect our use of all of Gray et al.’s (2007) descriptions, rather than only a subset focused on drive states or basic emotions, as well as our use of face stimuli drawn from the infant literature, which together may have more comprehensively engaged participants’ judgments. In addition, our use of a two-alternative forced-choice task, rather than a single Likert-scale rating, may have made the critical alternative more explicit and thereby clarified what the task was intended to probe.

Our findings thus challenge the conclusions from Khalid et al. (2016) that gaze direction is used as a cue for understanding intentional mental states primarily. The literature on infants’ intuitive use of gaze direction also suggests an alternative. Adults may intuitively link descriptions of either phenomenal or intentional mental states to straight-ahead gaze, the former interpreting the straight-ahead gaze as social (“at-me”) and the latter as indicating general visible accessibility for goal-directed action. Unlike for straight-ahead gaze, there may be no such intuitions, neither phenomenal nor intentional, for judgments of averted gaze. The link between phenomenality descriptions and averted gaze may instead arise and become dominant in adults only after learning that certain phenomenal contexts involve inner experiences associated with averted gaze. This learning may start with the connections young children begin to make between others’ perceptions and their inner experiences (e.g., Wellman et al., 2000) and might also rely in part on an understanding of the relevant mental-state language that can communicate such contexts explicitly (Astington & Baird, 2005; de Villiers & de Villiers, 2014; Harris et al., 2005).

We tested this interpretation by presenting children with the same task as adults during an age window in which there is developmental change in their judgments of descriptions of others’ mental-state capacities (like those used in Gray et al., 2007; Weisman et al., 2018), in their mental-state and theory-of-mind reasoning (Grazzani & Ornaghi, 2012; Harris et al., 2005; Pons et al., 2003), and in their mental-state-language understanding (Baron-Cohen et al., 2010). Unlike adults, children chose the straight-ahead-gaze face more for both the indirect phenomenality and indirect intentionality descriptions, and this effect did not change with age. For the direct descriptions, children chose the straight-ahead-gaze face more for the intentionality description, but they chose at chance for the direct phenomenality description. This latter effect did change with age: The oldest children showed adult-like choices of the averted-gaze face for the direct phenomenality description.

Children might thus indeed still be linking both phenomenal and intentional mental-state descriptions to straight-ahead gaze, reflecting early emerging, intuitive links between gaze direction and mental-state understanding. Only by around age 8 years of age are children beginning to show new judgments that link phenomenal mental-state descriptions to averted gaze, like adults do. This link between phenomenal mental-state descriptions and averted gaze might be learned through experience with certain phenomenal contexts and rely at least somewhat on children’s developing understanding of the mental-state language that can communicate such contexts explicitly.

### Conclusion

The content on which our mature social concepts are built may be characterized within the framework of our early emerging sensitivities to phenomenal social beings and intentional agents as well as to the language that engages such early intuitions. Language might reinforce these prelinguistic intuitions or change them by evoking particular contexts and may do so only later in development and after extensive learning. If, in adulthood, our social concepts have indeed been built on such prelinguistic intuitions and language, then these intuitions may continue to affect our mature, linguistically driven social reasoning, including our definitions of personhood (Dillon, 2024), our judgments in moral dilemmas (Knobe & Prinz, 2008), and even entire systems of ethics and their inevitable, intractable aporias (Reilly, 2019; Sinnott-Armstrong, 1988).

## ACKNOWLEDGMENTS

We thank Gillian Broome, Cindy Luo, Deisy Martinez, and Eli Mitnick for their assistance in data collection, and Rida Saad for her assistance in recruiting participants. We thank Brian Reilly for the implementation of the readability analysis and comments on the manuscript. We thank all participating families.

## FUNDING INFORMATION

This work was supported in part by a New York University Mega Grants Initiative Seed Fund to M.R.D.

## AUTHOR CONTRIBUTIONS

M.M.: Conceptualization; Data curation; Formal analysis; Methodology; Project administration; Validation; Visualization; Writing – original draft; Writing – review & editing. M.R.D.: Conceptualization; Funding acquisition; Methodology; Resources; Supervision; Visualization; Writing – original draft; Writing – review & editing.

## CODE AND DATA AVAILABILITY STATEMENTS

Experiment 1 was not preregistered, but Experiments 2–4 were preregistered prior to data collection.

The preregistration for Experiment 2 is available here: https://osf.io/fqh74/.

The preregistration for Experiment 3 is available here: https://osf.io/xf65e/.

The preregistration for Experiment 4 is available here: https://osf.io/z5yq8/.

The materials, methods, data, analysis code, and model outputs for all experiments are available here: https://osf.io/rztc6/.

## Supplementary Material


