# Supplementary material for: Descriptions of Phenomenal Versus Intentional Mental States Are Differentially Associated With Gaze Direction
Source: Open Mind (Camb). 2026 Sep 20;10:1550–66. doi: 10.1162/OPMI.a.386 (PMC13733551; doi:10.1162/OPMI.a.386)
Supplement: Supplementary file 1 [file opmi-10-1550-s001.pdf]

Descriptions of phenomenal versus intentional mental states are differentially associated with gaze direction

Supplementary Methods

Table 1A provides the indirect mental-state descriptions adapted from Gray et al. (2007), and **Table 1B** provides the direct mental-state descriptions adapted from Spelke (2022). For the indirect descriptions, we also show Gray et al.'s (2007) factor loadings on to their "experience" and "agency" dimensions from their principal components analysis. We treat the descriptions that loaded higher onto their experience component as indirect phenomenality descriptions, and the descriptions that loaded higher onto their agency component as indirect intentionality descriptions. Because the descriptions within a category also varied by the magnitude of their factor loadings on to each component, we also consider this variation in our planned analyses (see the main text).

Table 2 provides the glosses for the indirect descriptions available to children in **Experiment 4.**

Supplemental Results

Table 2 provides the number of glosses children requested for the indirect descriptions, with age broken down in years (6-, 7-, or 8-year-olds). Fifty-eight children requested at least one gloss for the indirect descriptions, including a total of 113 requests, 66 for indirect phenomenality descriptions and 47 for indirect intentionality descriptions. Since there were 11 indirect phenomenality descriptions but only 7 indirect intentionality descriptions, we compared children's requests for glosses across the two description types using a binomial test setting the

test proportion at .61, which is the number of indirect phenomenality descriptions (11) divided by the total number of descriptions (18). We compared this proportion to .58, which is the number of requests for phenomenality glosses (66) divided by the total number of requests for glosses (113). The test was not significant (p = .564), providing no evidence that the number of requests children made for glosses differed by indirect description type. Children's requests were nevertheless more evenly distributed across the indirect phenomenality descriptions versus the indirect intentionality descriptions, and older versus younger children seemed to request more glosses specifically for the phenomenality descriptions (see **SI**). For the direct descriptions, there was one request for the phenomenality description gloss and 6 requests for the intentionality description gloss.

Table 1A

Indirect descriptions of mental states, adapted from Gray et al. (2007), and their factor loadings.

Mental State	Description	Experience	Agency
Hunger	This character is capable of feeling hungry.	**0.97**	0.15
Fear	This character is capable of feeling afraid or fearful.	**0.93**	0.31
Pain	This character is capable of experiencing physical or emotional pain.	**0.89**	0.41
Pleasure	This character is capable of experiencing physical or emotional pleasure.	**0.85**	0.51
Rage	This character is capable of experiencing violent or uncontrolled anger.	**0.78**	0.58
Joy	This character is capable of experiencing joy.	**0.67**	0.6
Desire	This character is capable of longing or hoping for things.	**0.76**	0.64
Embarrassment	This character is capable of experiencing embarrassment.	**0.7**	0.65
Personality	This character is capable of having personality traits that make them unique from others.	**0.72**	0.68
Consciousness	This character is capable of having experiences and being aware of things.	**0.71**	0.69
Pride	This character is capable of experiencing pride.	**0.71**	0.69
Thought	This character is capable of thinking.	0.68	**0.73**
Communication	This character is capable of conveying thoughts or feelings to others.	0.66	**0.74**
Planning	This character is capable of making plans and working towards goals.	0.55	**0.82**
Emotion Recognition	This character is capable of understanding how others are feeling.	0.54	**0.83**
Memory	This character is capable of remembering things.	0.33	**0.91**
Morality	This character is capable of telling right from wrong and trying to do the right thing.	0.35	**0.93**
Self-control	This character is capable of exercising self-restraint over desires, emotions, or impulses.	0.18	**0.97**

Table 1B.

Direct descriptions of mental states, adapted from Spelke (2022).

Phenomenal	This character is a social being who has emotions and feelings like you do.
Intentional	This character is an agent who has intentions and pursues goals efficiently.

Table 3.

Glosses available to children in ***Experiment 4****.*

Mental State	Description	Gloss	6	7	8
Hunger	This character is capable of feeling hungry.	They can feel hungry.	0	0	1
Fear	This character is capable of feeling afraid or fearful.	They can feel scared.	1	0	0
Pain	This character is capable of experiencing physical or emotional pain.	They can get hurt or feel bad.	3	2	3
Pleasure	This character is capable of experiencing physical or emotional pleasure.	They can feel good.	5	6	4
Rage	This character is capable of experiencing violent or uncontrolled anger.	They can feel very angry.	2	2	2
Joy	This character is capable of experiencing joy.	They can feel very happy.	1	0	0
Desire	This character is capable of longing or hoping for things.	They can want things.	3	2	1
Embarrassment	This character is capable of experiencing embarrassment.	They can feel embarrassed.	1	2	0
Personality	This character is capable of having personality traits that make them unique from others.	They can think, feel, and behave differently from others.	2	1	5
Consciousness	This character is capable of having experiences and being aware of things.	They can experience what's going on around them.	0	1	2
Pride	This character is capable of experiencing pride.	They can feel good about good things they do.	5	4	5
Thought	This character is capable of thinking.	They can think about things.	0	0	0
Communication	This character is capable of conveying thoughts or feelings to others.	They can show other people about how they feel.	1	6	9

Planning	This character is capable of making plans and working towards goals.	They can plan how to get what they want.	1	0	0
Emotion Recognition	This character is capable of understanding how others are feeling.	They can understand other people’s feelings.	0	1	0
Memory	This character is capable of remembering things.	They can remember things.	0	0	0
Morality	This character is capable of telling right from wrong and trying to do the right thing.	They can tell right from wrong and try to do the right thing.	0	0	2
Self-control	This character is capable of exercising self-restraint over desires, emotions, or impulses.	They can be patient and calm when they’re upset.	8	10	9
Social Being	This character is a social being who has emotions and feelings like you do.	They have emotions and feelings like you do.	0	1	0
Agent	This character is an agent who has intentions and pursues goals efficiently.	They want things and try their best to get those things.	0	5	1

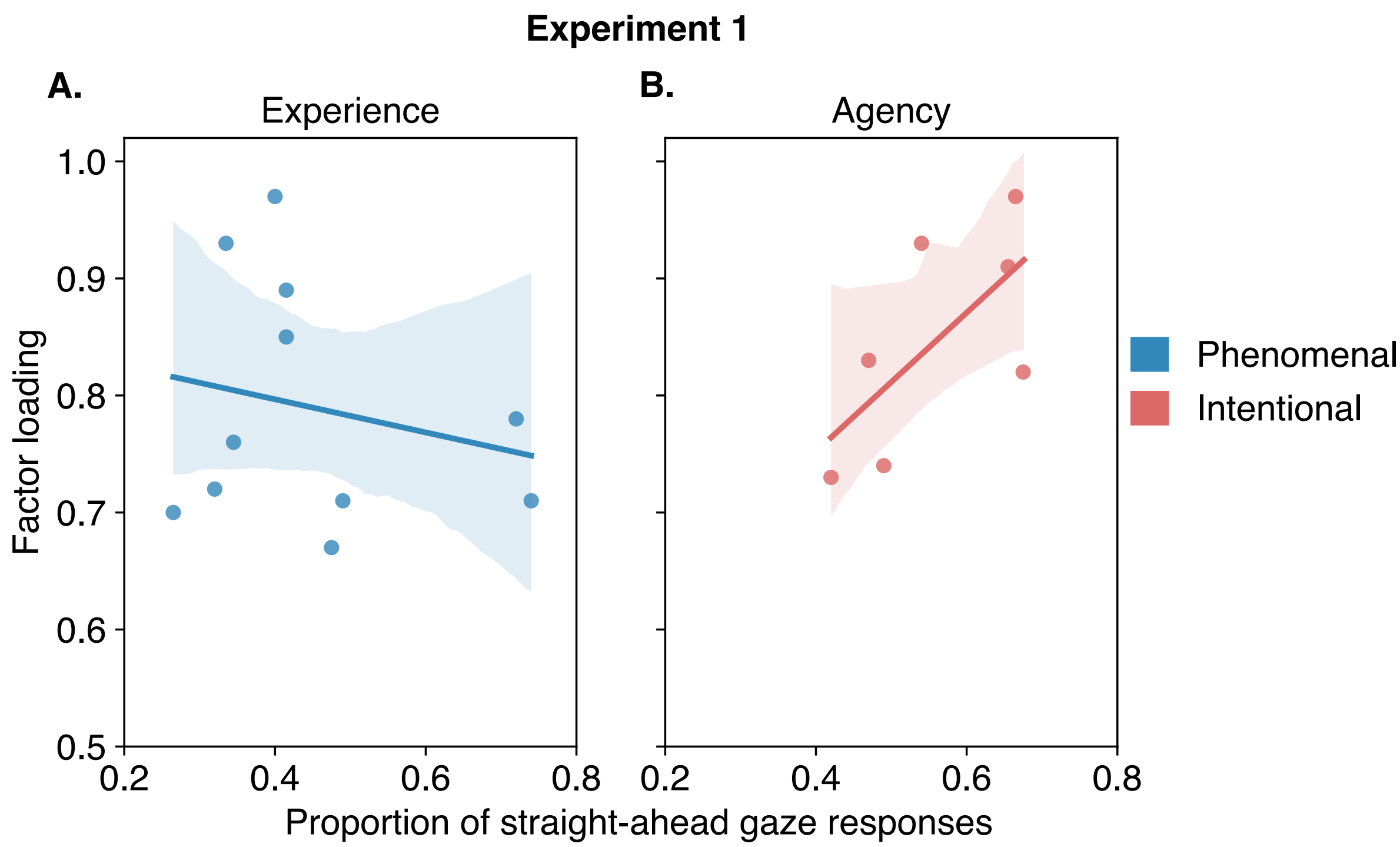

Figure S1. The factor loadings onto the experience and agency dimensions of Gray et al. (2007) predicted by the proportion of straight-ahead-gaze responses from **Experiment 1**. Results are shown for the (**A**) the phenomenality descriptions and experience dimension and the (**B**) the intentionality descriptions and agency dimension. See the main text for the relevant statistical reporting.

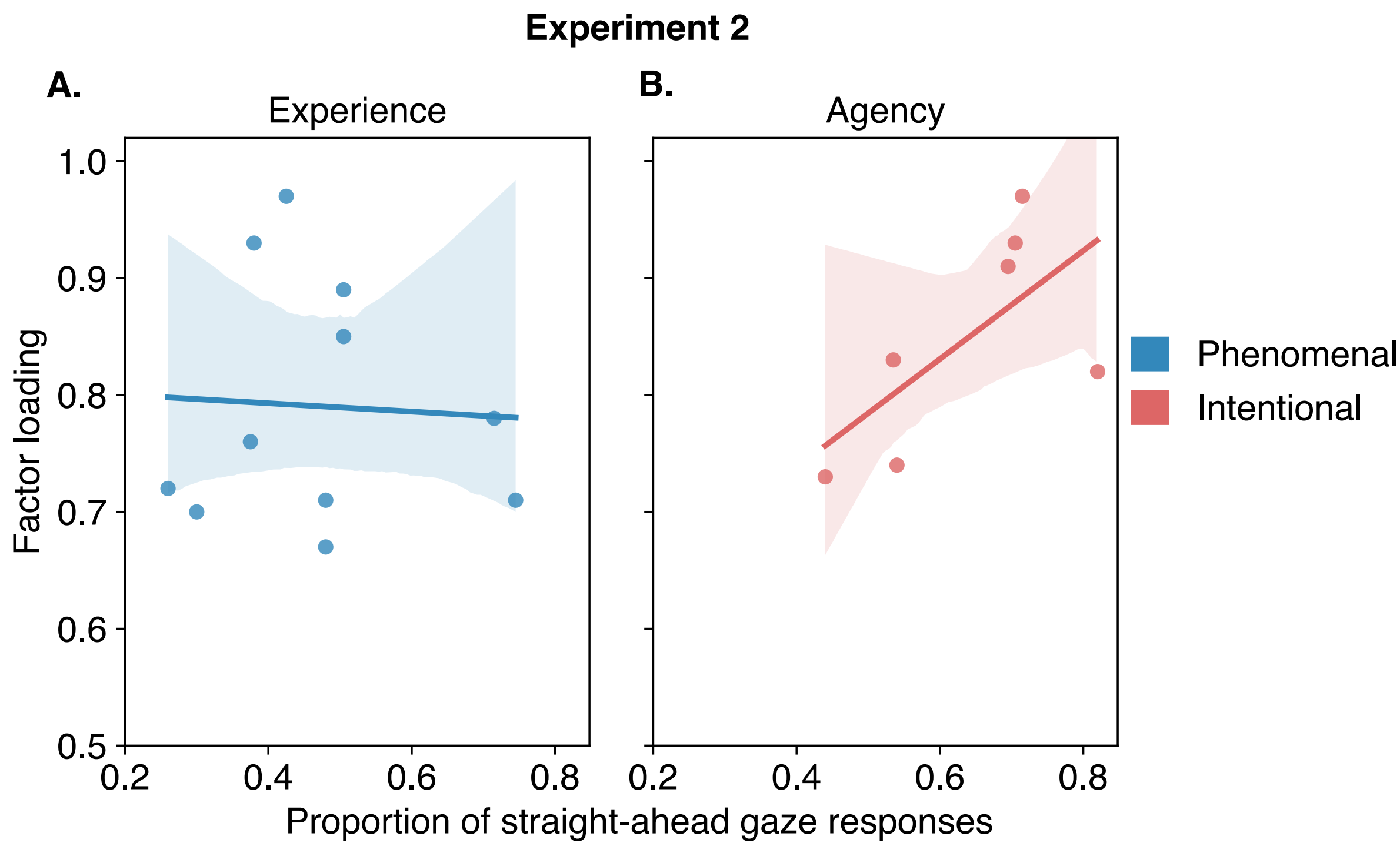

Figure S2. The factor loadings onto the experience and agency dimensions of Gray et al. (2007) predicted by the proportion of straight-ahead-gaze responses from **Experiment 2**. Results are shown for the (**A**) the phenomenality descriptions and experience dimension and the (**B**) the intentionality descriptions and agency dimension. See the main text for the relevant statistical reporting.

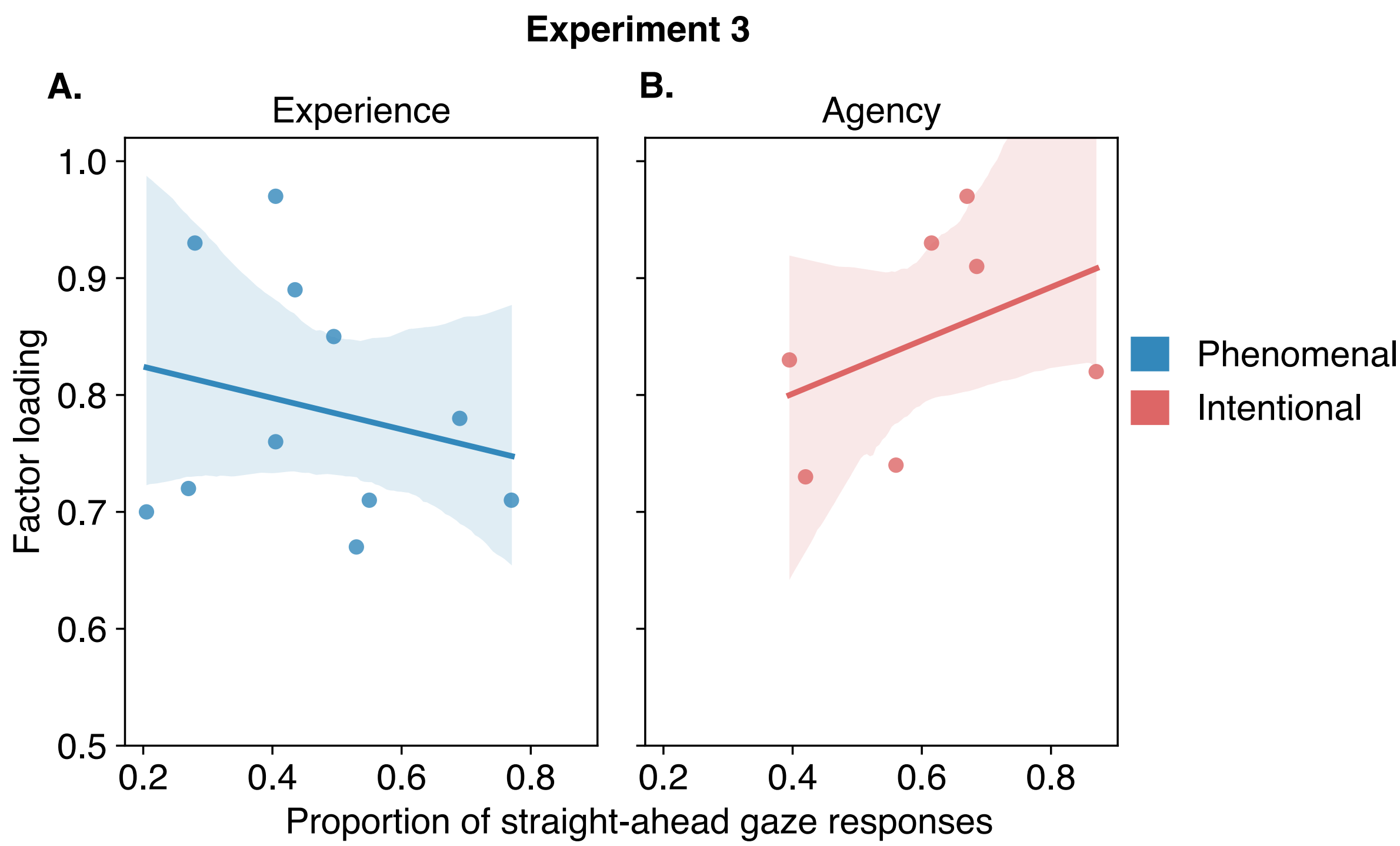

Figure S3. The factor loadings onto the experience and agency dimensions of Gray et al. (2007) predicted by the proportion of straight-ahead-gaze responses from **Experiment 3**. Results are shown for the (**A**) the phenomenality descriptions and experience dimension and the (**B**) the intentionality descriptions and agency dimension. See the main text for the relevant statistical reporting.

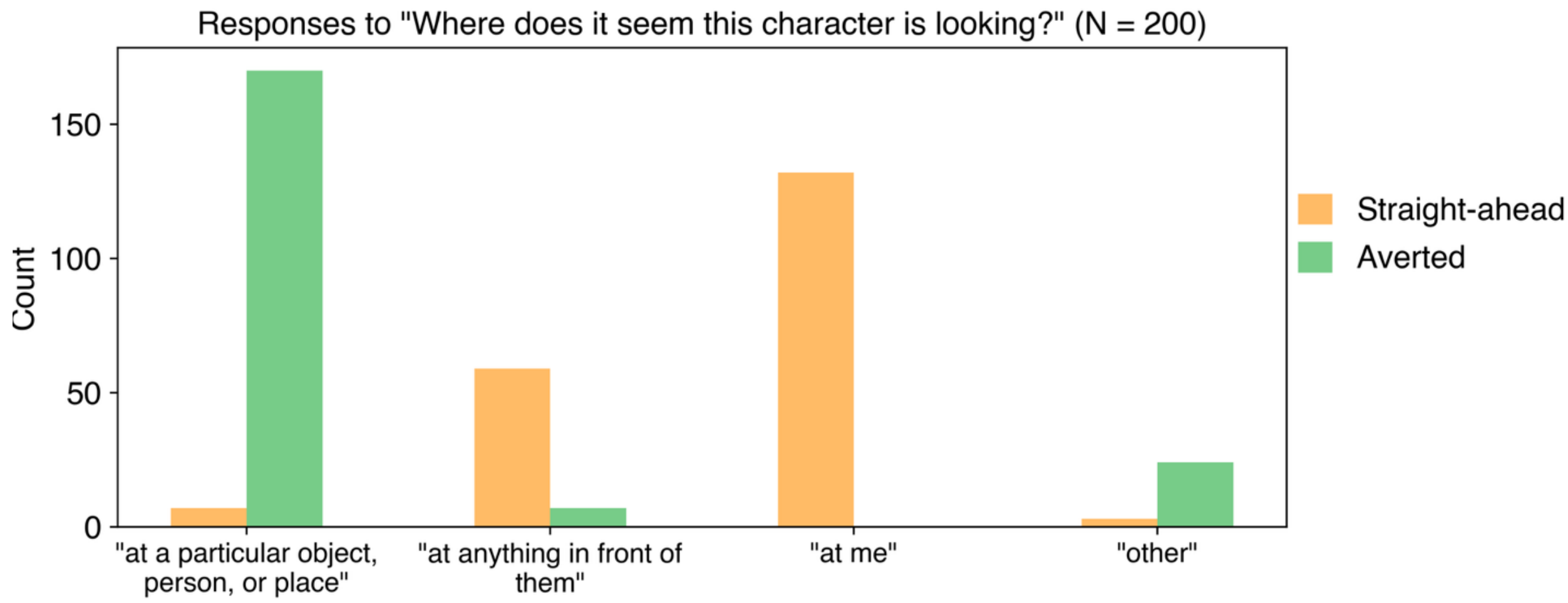

Figure S4. Raw counts of adults' responses to the two debriefing questions that asked them where they thought each of the two face pictures they saw (averted gaze and straight-ahead gaze) were looking in **Experiment 3**. Participants responded to these questions in a random order and after responding to all of the indirect and direct descriptions.

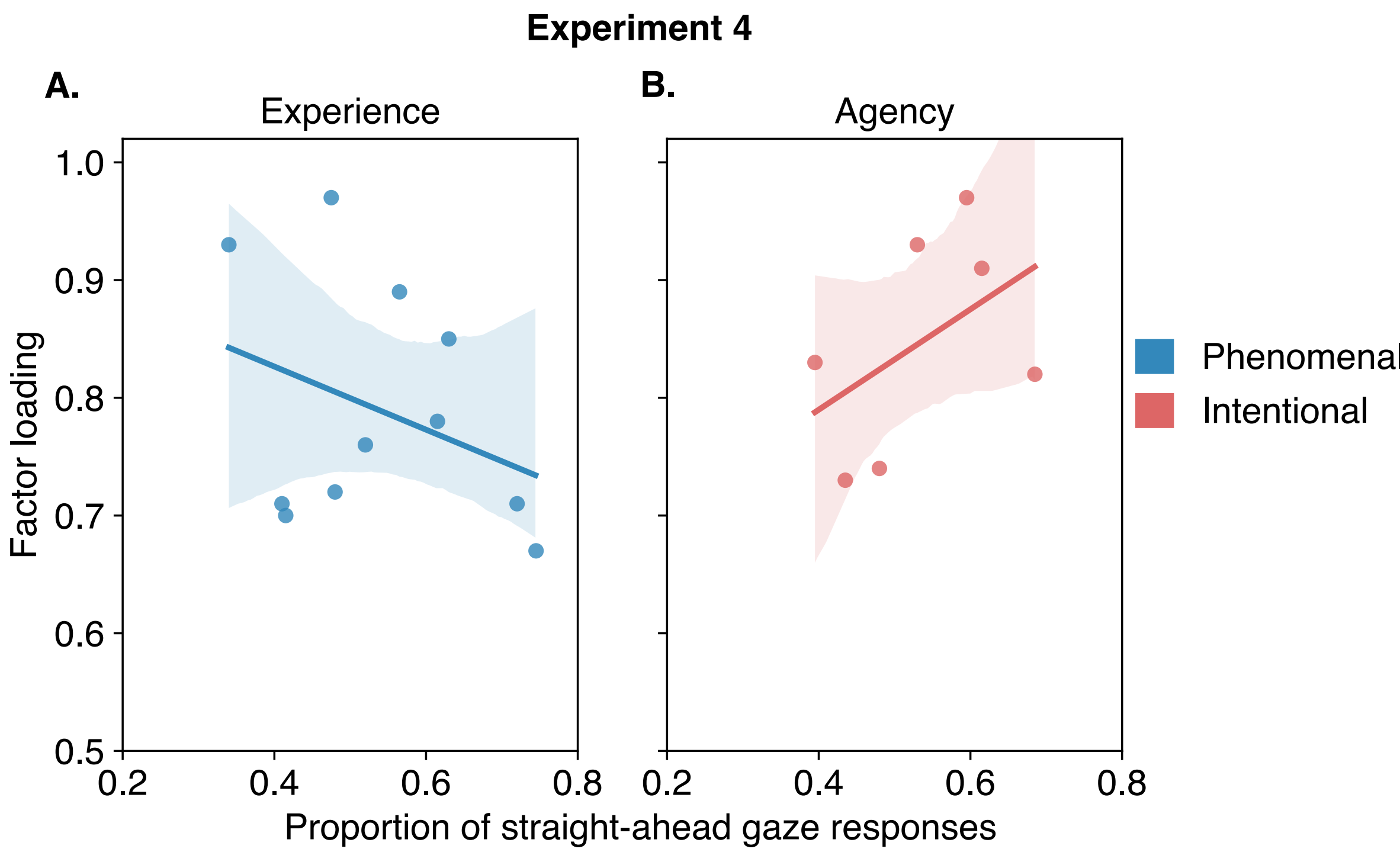

Figure S5. The factor loadings onto the experience and agency dimensions of Gray et al. (2007) predicted by the proportion of straight-ahead-gaze responses from **Experiment 4**. Results are shown for the (**A**) the phenomenality descriptions and experience dimension and the (**B**) the intentionality descriptions and agency dimension. See the main text for the relevant statistical reporting.
